# Not seeing the trees for the forest. The impact of neighbours on graph-based configurations in histopathology

**DOI:** 10.1186/s12859-024-06007-x

**Published:** 2025-01-11

**Authors:** Olga Fourkioti, Matt De Vries, Reed Naidoo, Chris Bakal

**Affiliations:** 1 The Institute of Cancer Research, London, United Kingdom; 2 Imperial College, London, United Kingdom

**Keywords:** Computational pathology, Graph-neural networks, Visualization, Attention, Context, Cell classification

## Abstract

**Background:**

Deep learning (DL) has set new standards in cancer diagnosis, significantly enhancing the accuracy of automated classification of whole slide images (WSIs) derived from biopsied tissue samples. To enable DL models to process these large images, WSIs are typically divided into thousands of smaller tiles, each containing 10–50 cells. Multiple Instance Learning (MIL) is a commonly used approach, where WSIs are treated as bags comprising numerous tiles (instances) and only bag-level labels are provided during training. The model learns from these broad labels to extract more detailed, instance-level insights. However, biopsied sections often exhibit high intra- and inter-phenotypic heterogeneity, presenting a significant challenge for classification. To address this, many graph-based methods have been proposed, where each WSI is represented as a graph with tiles as nodes and edges defined by specific spatial relationships.

**Results:**

In this study, we investigate how different graph configurations, varying in connectivity and neighborhood structure, affect the performance of MIL models. We developed a novel pipeline, K-MIL, to evaluate the impact of contextual information on cell classification performance. By incorporating neighboring tiles into the analysis, we examined whether contextual information improves or impairs the network’s ability to identify patterns and features critical for accurate classification. Our experiments were conducted on two datasets: COLON cancer and UCSB datasets.

**Conclusions:**

Our results indicate that while incorporating more spatial context information generally improves model accuracy at both the bag and tile levels, the improvement at the tile level is not linear. In some instances, increasing spatial context leads to misclassification, suggesting that more context is not always beneficial. This finding highlights the need for careful consideration when incorporating spatial context information in digital pathology classification tasks.

**Supplementary Information:**

The online version contains supplementary material available at 10.1186/s12859-024-06007-x.

## Background

In recent years the advent of deep learning (DL) has paved the way for the establishment of digital pathology as a vital tool in modern pathology [1–10]. However, while histopathological images are relatively easy to obtain, deliberately labelling each pixel in every mega-/giga-pixel Whole Slide Image (WSI) with expert-based ground-truth descriptions can be prohibitively time-consuming. The need to handle this partially or ambiguously labelled training data gave rise to a novel paradigm in machine learning, weakly supervised learning (WSL) [11].

Multiple Instance Learning (MIL) is a type of WSL where the training examples are arranged in sets of labelled bags, each containing unlabelled instances. In the case of of digital pathology, these instances are ’tiles’, or sub-regions of the WSI. Using this weakly labelled training data, MIL aims at learning a model capable of correctly classifying both new bags and new instances [12, 13]. MIL is particularly useful to image-based pathology classification due to its ability to reason on subsets of data; a computational necessity when analyzing very large images [14].

In the context of using MIL in cancer histopathology, the task is to determine if the tissue imaged in the WSI (‘bag’) can be considered to have cancerous sub-regions (tumour cells), or incidences - amongst expected incidences of non-cancerous regions (normal cells) [15–19]. Typically, learning is accomplished by identifying commonalities between cancerous instances across WSIs. However, because of the high degree of inter-tumour heterogeneity, as well as the histologic overlap between cancer and other neoplasms, automatically learning common morphological signatures of cancerous tissue in WSIs remains a challenging problem [20]. Additionally, because there is extensive intra-tumour heterogeneity in cancer cells, the instances within a positive bag can differ substantially and not all of them equally affect the final diagnosis i.e., cancer or not [21]. Therefore, the consideration of an instance’s location in a WSI is particularly relevant to using MIL in histopathology; where the cells are not distributed independently inside an image but there are underlying patterns governing their spatial arrangement. As an example, pieces of a jigsaw puzzle contain spatial information as to their position in the completed puzzle and the identity of their neighbours. Similarly, location and contextual information of cancerous tissues can be leveraged to learn whether an instance is cancerous because it neighbours another region with high probability of being cancerous.

In conventional MIL problems using context-based approaches, it’s common to construct a graph using a fixed number of nodes [22–24]. Despite this common practice, there hasn’t been a specific study investigating the influence of the number of graph nodes on the overall performance of a MIL model. Recognizing the significance of how these graphs are constructed and the critical role of selecting which elements to include for the graph construction, here we developed a simple neural network architecture to investigate the impact of different graph configurations on the overall model performance. Specifically, we represent each cell as a node of a graph, which is connected to other nodes in the image based on spatial proximity and feature similarity. Nodes that are spatially adjacent and morphologically close are linked by an edge. Based on those criteria, we construct an adjacency matrix which operates as a mask that enables us to attend over each tile and its surrounding nodes, calculate their attention coefficients, and produce an average attention score for each node of the graph. We then progressively expand the pool of nodes that can be connected, examining how extending the range of connectivity affects the model’s effectiveness. The main goal of the proposed architecture is to generate a flexible descriptor, capable of capturing the contextual information of each node, allowing for a thorough analysis of various graph configurations. Finally, we demonstrate the attention maps generated for different graph configurations and investigate how the choice of the number of nodes affects the produced visualisations.

## Methods

### Problem Definition:

We assume a training set consisting of WSIs: $X = \{X_1, X_2, X_3,... X_m \}$ and their associated labels $Y = \{Y_1, Y_2, Y_3,... Y_m \}$, where $Y_i \in \{0,1\}$. We further assume that for every bag we are given a set of instances $X_i = \{x_{i_1}, x_{i_2}, x_{i_3},... x_{i_n} \}$. Every instance is also associated with a label $y_{i_j} \in Y_i$. However, these labels remain unknown during the training stage. According to the typical MIL definition, a bag is labeled negative if it contains only negative instances, while the presence of at least one positive instance is sufficient and necessary evidence to label it positive. Using the max operator this statement can be re-formulated in the following form:

1 $$\begin{aligned} Y_i= \max (y_{i_j}) \end{aligned}$$

The lack of differentiablility of the maximum based objective makes it unsuitable for bag level classifiers. However, in their work ([25]) have proved that a MIL model can be trained instead by optimizing binary cross entropy which according to ([26]) can be expressed as:

2 $$\begin{aligned} L = -\frac{1}{N}\sum _{i=1}^N Y_i\log (\hat{Y_i})+(1-Y_i)\log (1-\hat{Y_i}) \end{aligned}$$

where $\hat{Y_i}$ refers to the score of the bag label.

### K-MIL model

**Fig. 1:**
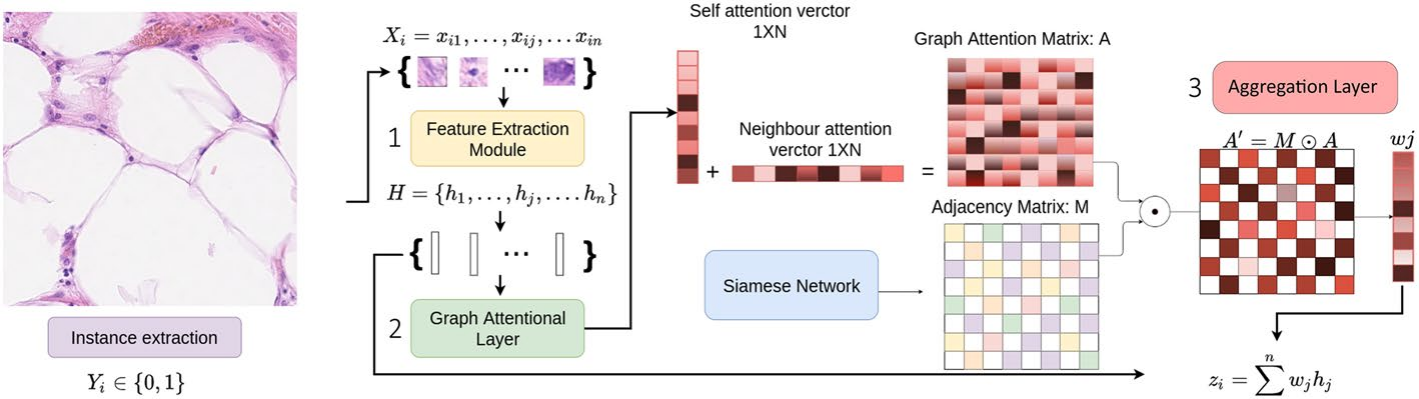
Components of K-MIL model: 1) a feature extraction module that receives a set of tiles ${x_i}$ as input and outputs a set of hidden representations h_i_ 2) a graph attentional layer responsible for outputting a graph attention NxN matrix and 3) a layer that enables the aggregation of the attention coefficients of the neighbourhood of each instance through an adjacency matrix

To quantify the role of context on classification performance we first developed a pipeline for cell classification; K-MIL. While this is a new model, its primary purpose is to enable a systematic investigation of various graph configurations in the context of histopathology. To ensure a fair comparison with existing approaches, we designed our model to closely align with a standard attention pooling framework, with the inclusion of additional layers that provide context-specific information. Importantly, our model deliberately avoids the use of more sophisticated layers or mechanisms (e.g., multi-head attention, deeper networks) that could introduce confounding factors, making it difficult to isolate the impact of context. The approach is described in Fig. 1. It can be decomposed into the following three components i) the feature extraction module, which consists of a stack of convolutional and max pooling layers as well as dense layers responsible for transforming the original patch input to a low dimensional feature representation, ii) an attention mechanism responsible for outputting an attention NxN matrix and iii) a neighbour layer which is a permutation invariant pooling operator that enables the aggregation of the attention coefficients produced in the previous step through an NxN adjacency matrix.

**Attention Matrix**

Each instance of the bag is processed by a backbone network, to produce a set of hidden representations $H= \{h_1,..., h_i,....h_n\}$, $hi \in R_F$.

To calculate the attention weights among the instances, we propose to use a single attentional layer that implicitly specifies a different weight between any pair of instances in the bag connected or not. The aforementioned attention mechanism is parametrized by neural networks. Notably, the attentional setup follows closely the work of ([27]). The main difference between the two lies in: a) the activation function used, which in our case is tanh to ensure that the output values of the attention matrix will be constrained into a small value range and b) in our case the attention coefficients are used to compute a single average score across the different neighbours of a node, whereas in the work of ([27]) the attention coefficients used to compute a linear combination of the features corresponding to them.

Given the set of hidden representations for every instance obtained from the backbone model $H= \{h_1, h_2, h_3,....h_n\}$, $hi \in R_F$, the output of the attentional layer is an attention matrix $A_{n,n}$. Every element $a_{i,j}$ of the matrix$A_{n,n}$ corresponds to an attention coefficient which serves as an indicator of the influence of the instance i to instance j:

3 $$\begin{aligned} \alpha _{ij}= tanh (\vec {a}^T [W\vec {h_i}|| W\vec {h_j}]) \end{aligned}$$

**Adjacency Matrix**

To formulate the neighbourhood prior and leverage the pairwise relations among the instances of a bag we resort to an adjacency matrix $M_{n,n}$ which indicates the presence or not of a link between every instance in the bag and the rest. The attention coefficients should be computed only for the instances that belong to the neighbourhood of the target instance $n_i$. That way the adjacency matrix operates as a mask, which when multiplied element-wise with the attention matrix computed in the previous step, ensures that only the neighbouring values of an instance will be preserved and the rest will be discarded.

4 $$\begin{aligned} A' = M \odot A \end{aligned}$$

### Computing the adjacency matrix

A naturally arising question is which criteria should be applied to best describe an instance’s neighbourhood. In our implementation, we take into account two different distance metrics to construct the adjacency matrix: one based on the euclidean distance and the other based on the siamese distance.

As the position of the instances in the bag is either known a priori or can be inferred in the case they are cropped, we can construct the adjacency matrix using the Euclidean distance between the spatial locations of the instances to determine the existence of a connection between two instances $x_i$ and $x_j$, such as:

5 $$\begin{aligned} M_{i_j} = \left\{ \begin{array}{ccc} 0, & otherwise\\ 1, & \hbox { if}\ i \in KNN(j) \end{array} \right. \end{aligned}$$

where KNN refers to the K nearest neighbour to the patch i.

The Euclidean distance can capture the spatial relationships between neighbouring instances, but cannot model more complex relationships. Siamese nets on the other hand can be trained to discover dynamically and adaptively which instances are relevant to each other ([28]).

Typically, they are trained in a supervised manner in a collection of positive and negative pairs ([28]) enabling the network to learn how similar two images are to one another. Features of similar image pairs are encouraged to be closer together in the feature space, and dissimilar ones far away from each other.

In situations where labeled training data is unavailable, siamese networks offer a solution by being trained in an unsupervised manner. This approach utilizes a training set constructed from naive nearest neighbour relations, as demonstrated in the work of ([29]). The concept behind these naive nearest neighbour relations is grounded in the idea that instances in close proximity tend to share more similar morphological characteristics. In our training process, positive pairs are formed by pairing each instance with its spatially closest counterparts within a manually defined radius (r), while negative pairs are created by randomly sampling an equal number of non-neighbouring instances. The network then learns these intricate neighbouring relations by optimizing the contrastive loss:

$$L(\theta ;z_i. z_j) = \left\{ \begin{array}{ll} \left\| z_i - z_j\right\| ^2 \\ (z_i, z_j) \text { is a positive pair};\\ max{(m-\left\| z_i - z_j \right\| ^2 ,0)}^2 \\ (z_i,z_j) \text {is a negative pair }.\end{array} \right.$$

where $z_i, z_j$ are the feature representations corresponding to the ith, jth images and m is a margin.

Once trained on this subset of images, the pre-trained siamese net is integrated to our model to output a distance metric d between every instance in a bag and its k closest neighbouring instances independently of any radius. The adjacency matrix is constructed as follows:

6 $$\begin{aligned} M_{i_j} = \left\{ \begin{array}{ccc} 0, & \text {otherwise}\\ exp(-d), & \hbox { if}\ i \in KNN(j) \end{array} \right. \end{aligned}$$

where KNN refers to the k nearest neighbour to the patch i and d to the distance learnt by the Siamese net.

### Final attention score

After obtaining the attention coefficients that correspond to the neighbours of every instance, the last step is to aggregate the contextual information into a single attention weight for each instance. There are several operators that can be used to perform feature aggregation. In our experiments, we utilize the mean operator, followed by a softmax function to ensure that all weights sum up to one:

7 $$\begin{aligned} w_i= \frac{\exp ({\frac{1}{K}\sum _{j \in n_i} \alpha _{ij}})}{\sum _{k=1}^{N}\exp ({\frac{1}{K} \sum _{j \in n_k} \alpha _{kj}})} \end{aligned}$$

where N refers to the number of instances present in each bag, $n_i$ to the neighbours of every instance i and K to the number of nearest neighbours.

Lastly the updated weights are multiplied in an element-wise fashion with their corresponding bag embeddings $H={h_1,h_2,...., h_n}$ as follows:

8 $$\begin{aligned} z_i= \sum _{i=1}^{N} w_i {h_i} \end{aligned}$$

### Measuring the effect of context

By adjusting the number of nodes in the model, we investigate the effects of various graph configurations on the overall performance and the attention maps produced. Our model has two different variations: Euclidean and Siamese. The euclidean version uses spatial information to define contextual relationships, while the siamese version additionally considers feature similarity to establish connections between instances. To understand the impact of contextual information on cell classification tasks, we compare these two variations against non-context models that treat each instance independently.

### Context models

Context models are divided into two distinct categories: the **euclidean** version and the **siamese** version.

- The **euclidean** version forms the adjacency matrix based purely on spatial criteria, using the coordinates of the tiles to establish contextual relationships. In this version, two nodes are connected if they are spatially adjacent, and the value in the adjacency matrix is set to 1.
- The **siamese** version apart from the spatial criteria introduces similarity-based constraints. This allows the model to establish edges in the graph not just based on how close the individual tiles (nodes) are in space, but also by considering how similar they are in terms of their features or patterns. In this version, the value in the adjacency matrix is determined by the similarity between the feature representations of spatially adjacent nodes.

Furthermore, to showcase the ability of our model to efficiently leverage contextual information we also set one additional baseline: **Random K-MIL** which creates edges between different nodes randomly.

### Non-context models

Before performing a systematic investigation on how context can affect classification, we first compared our simple neighbour pooling strategy to other MIL pooling methods successfully deployed in the past. One of them is the embedding approach of ([30]) which implements attention as a function of the features of each instance alone. For clarity of notation, we refer to this model as **ABMIL** and to the version of it that makes uses of the gated attention mechanism as **gated ABMIL** [31]. We also compare our model to the **MI-NET** model and its variants **MI-NET with DS ** (deep supervision), **MI-NET with RC** (residual connection) [25]. MI-NET aims at learning a direct bag representation by utilising a specialised MIL pooling layer to aggregate input instances into a singular feature vector instead of inferring instance probabilities. There are three different pooling operators proposed. Here, we are using the max pooling operator as it demonstrates superior results. Additionally, **Mi-NET** which is the instance-based counterpart of the MI-NET is also taken into account.

### Datasets

We have conducted experiments on two histopathology datasets: the COLON cancer dataset and the USCB datasets.

The COLON cancer data set first presented in [32] includes 100 H&E stained histology images (bags) of colorectal adenocarcinomas, that were cropped from non-overlapping areas of 10 whole-slide images from 9 patients, at a pixel resolution of 0.55 $\upmu \hbox {m}$/pixel (20 $\times$ optical magnification). Every bag is composed of 27 x 27 sized nuclei that were manually annotated and belong in four different classes, i.e. epithelial, inflammatory, fibroblast and miscellaneous. For this dataset, our main focus is the detection of epithelial/normal cells. From a MIL perspective, this problem is formulated as follows: A bag is considered positive if it contains one or more nuclei belonging to the epithelial class, otherwise it is considered negative. From a clinical perspective correctly identifying epithelial cells can be highly relevant, since COLON cancer originates from epithelial cells ([33]).

The USCB dataset [34] contains 58 H&E strained image excerpts (26 malignant, 32 benign) from breast cancer patients. The initial size of the image is 896 x 768 pixels. Each image is represented as a collection of patches (32 $\times$ 32 pixels) and each patch contains a nucleus in the center and its adjacent tissues. For this dataset the objective is the detection of cancer cells.

## Results

### Context Improves the Detection of Epithelial-Cancer Cells

The WSI is represented as a graph where each cell is a node, and the edges between nodes are determined based on spatial criteria. Nodes are connected if the cells they represent are within a specific radius of each other. The distance between the cells must be below a certain threshold to form a connection, ensuring that each node is only connected to its neighbouring nodes within this predefined spatial range. By representing the WSI in this way, we can analyse the connectivity and relationships between cells, allowing for a detailed examination of the structure and behaviour of the WSI graph. For the rest of this section, we will refer the number of neighbours J=K-1, which provides a clearer and more intuitive description of the connectivity within each graph.

To evaluate the impact of contextual information on classification performance, we first analysed the accuracy of K-MIL models (euclidean and siamese) in the COLON cancer dataset, categorising WSIs as either ’epithelial-cancer containing’ or ’non-epithelial-cancer containing.’ These results are contrasted with the performance of non-contextual models, including Random-Net, ABMIL, and MiNET. In Table 1 we report the results when we create graphs with J=2 nodes.

Amongst the non-context models, RANDOM K-MIL, which when constructing the adjacency matrix creates edges between different nodes randomly, fails to produce meaningful results across both datasets. The MI-net model and its variants perform worse compared to our model, suggesting that there may be limitations in their ability to capture and leverage the underlying image structure effectively. ABMIL and gated ABMIL which incorporate attention mechanisms perform sufficiently well on both datasets. Specifically, the ABMIL models demonstrate competitive performance on par with the siamese version of the context models. Thus the accuracy of models that label WSIs as epithelial/non-epithelial is mildly improved when decisions are made by considering the context of each tile.

**Table 1:** Performance comparison of K-MIL against various baselines on the COLON cancer dataset comprising of H&E stained images.

METHOD	ACCURACY	PRECISION	RECALL	F-SCORE	AUC
5-Random net	0.781 ± 0.11	0.774 ± 0.16	0.799± 0.13	0.786±0.14	0.76±0.07
gated ABMIL net	0.905 ± 0.08	0.892 ± 0.15	0.911±0.10	0.898±0.18	0.985±0.03
ABMIL net	0.911 ± 0.08	0.921 ± 0.12	0.905±0.15	0.912±0.13	**0.987±0.02**
MI-NET	0.809 ± 0.129	0.841 ± 0.182	0.813±0.21	0.925± 0.02	0.925 ± 0.09
Mi-NET	0.842 ± 0.02	0.866 ± 0.01	0.816 ± 0.03	0.839 ± 0.02	0.914 ± 0.01
MI-NET with RC	0.879 ± 0.11	$0.820\pm$0.16	**0.950**±**0.15**	0.880±0.15	0.975±0.004
MI-NET with DS	0.853 ± 0.13	0.794 ± 0.27	0.853±0.28	0.822±0.27	0.959±0.07
Ours (euclidean)	0.909 ± 0.10	0.923 ± 0.12	0.925± 0.12	0.920±0.12	0.974±0.05
Ours (siamese)	**0.934**±**0.08**	**0.946**±**0.09**	0.930±0.13	**0.937**±**0.09**	**0.987**±**0.07**

The experiments were run 5 times and the average (± standard error of the mean) is reported. [bold]: Highlights the best-performing results in the respective metrics

### Classification of Epithelial-Cancer Cells

**Fig. 2:**
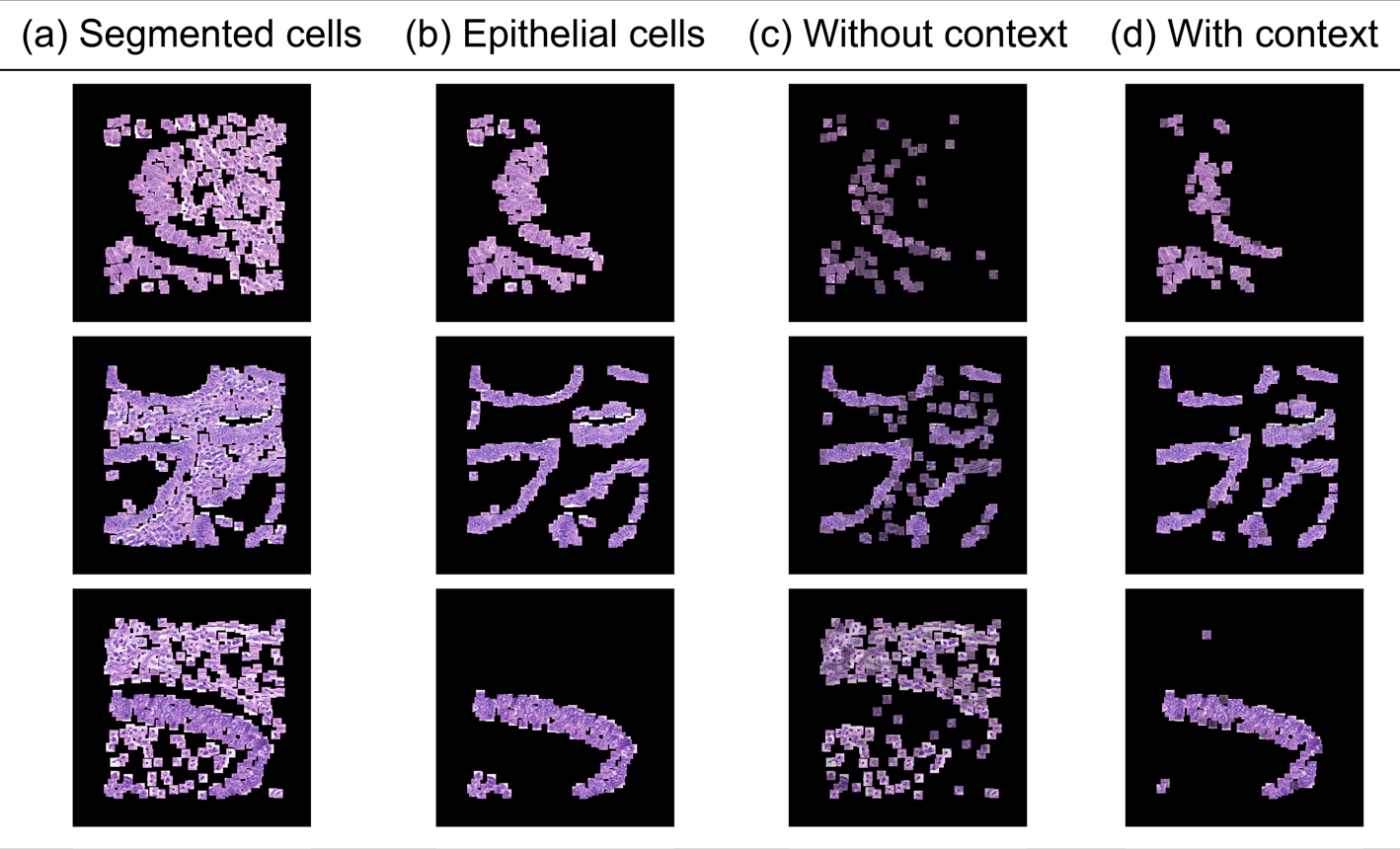
An example on the COLON cancer dataset highlighting how our method boosts instance-level classification accuracy qualitatively and quantitatively:** a** 27×27 segmented cells,** b** Ground truth cells,** c** Every patch multiplied by its corresponding attention weight using prior work [30],** d** Every patch multiplied by its corresponding attention weight using our model

In Fig. 2, we provide three representative examples of the attention maps produced by our model for the COLON dataset when our model achieves optimal performance (J=2). To provide a more comprehensive view of our results, we first display the extracted 27$\times$27 pixel patches centered around nuclei (Fig. 2a), which are the input to our models and represent cells belonging to the four different classes of the COLON cancer dataset. The figure then highlights patches containing epithelial cells which are the cells that our models are trying to identify (ground truth) (Fig. 2b). It further presents the patches, which have been identified as important by the ABMIL model (Fig. 2c). Patches with higher attention scores are made more prominent or visible, highlighting their significance or relevance according to the model. Finally, the salient patches detected by our model are presented in Fig. 2d. This layout enables a comparison between the different models, showcasing differences in how each model prioritises and values specific regions for understanding epithelial cell characteristics. In accordance with our quantitative analysis, we notice that the attention maps produced by our model (2d) tend to demonstrate a bigger overlap with Fig. 2b compared to ABMIL in Fig. 2c. In scenarios lacking contextual information, the model’s specificity is compromised, leading to numerous non-epithelial cells being misclassified as epithelial. In particular, the informative instances are selected based only on each instance’s feature representation, which is why they tend to appear scattered at random locations.

**Fig. 3:**
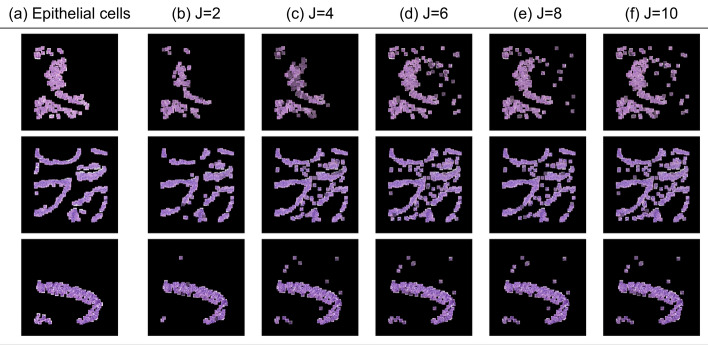
Attention map for different K on the COLON dataset:** a** ground truth cells,** b** J=2,** c** J=4,** d** J=6,** e** J=8,** f** J=10. Small variations of K do not lead to drastically different results. However, as K keeps ncreasing, the performance drops

**Fig. 4:**
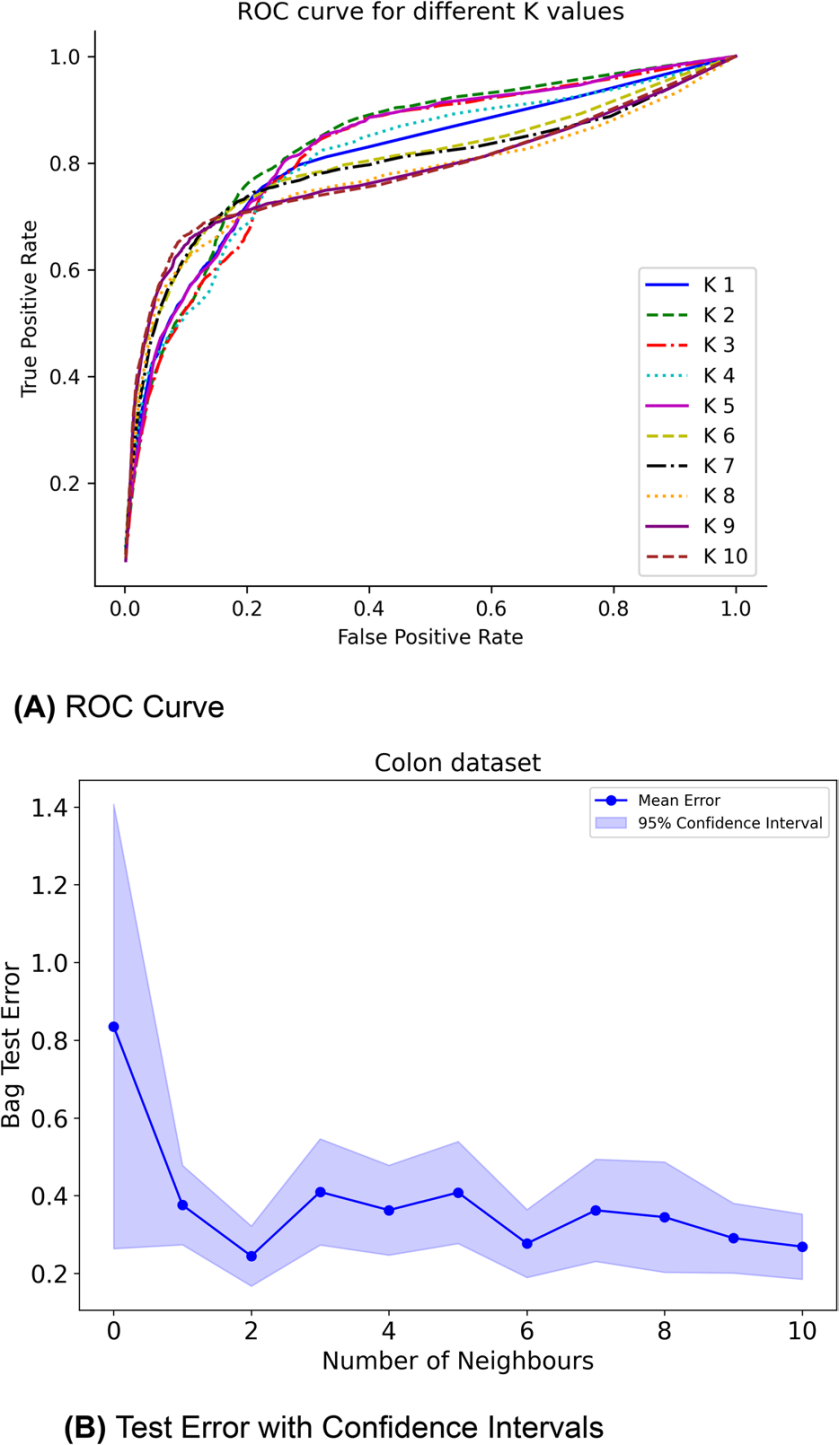
**a** ROC curve for the classification model performance.** b** Test error with respect to the number of nodes for the COLON cancer dataset. Each point on the plot represents the mean test error for a specific number of nodes, while the shaded regions around the points indicate the 95% confidence intervals

In Fig. 3, we present attention maps for different values of J. Notably, as the value of J increases, there is an increased overlap between the epithelial cells successfully recognised by our model and the ground truth. When J lies within the range of 2 to 6, the overlap between cells accurately identified by the model and the actual ground truth improves, indicating optimal performance in this range. However, a constant increase in the number of neighbours beyond this range leads to an increase in the number of false positives (cells that do not belong to the epithelial class but are recognised as such), yielding suboptimal results. Introducing more context into the analysis leads to an increase in the model’s sensitivity. However, beyond a certain point, this increase in sensitivity comes at the cost of decreased specificity, marked by a rise in false positives. This indicates a trade-off between sensitivity and specificity, where enhancing one can detrimentally affect the other. In addition to the qualitative evaluation, Fig. 4a displays the ROC curves for various graph configurations. The trend observed in the generated attention maps is confirmed in Fig. 3, indicating that configurations in the range 2 to 5 yield optimal results.

In Fig. 4b, the bag test error of our model with respect to the number of neighbours J is presented. Each point on the plot represents the mean test error for a specific number of nodes, while the shaded regions around the points indicate the 95% confidence intervals. Confidence intervals were computed using the normal approximation method based on the mean and standard deviation of the bag test error across multiple independently trained models (across 5 cross-validation folds). The standard deviation of the test error was used to calculate the standard error, which accounts for variability across the models. The intervals were derived by subtracting and adding the margin of error to the mean test error for each value of k (number of neighbors). Our first observation is that in the absence of contextual information the test error is significantly increased verifying once again the benefits from incorporating such information in our model. As the number of J increases, we observe a decline in test error, likely attributed to the supplementary information provided by the surrounding instances. When J varies between 2 to 5 we observe significant fluctuations in the test error likely attributed to the nature of the dataset. Specifically, the epithelial and non-epithelial cells form clusters that are tightly packed together. Therefore, small changes in J could change which clusters dominate the decision-making process for classifying a particular cell. Our second observation is that increasing the amount of context does not degrade the model’s performance at the bag level. However, as illustrated in Fig. 4a, adding more context negatively impacts the performance on an instance level.

### Classification of cancer cells

**Table 2:** Performance comparison of K-MIL against various baselines on the breast cancer dataset comprising of H&E stained images

METHOD	ACCURACY	PRECISION	RECALL	F-SCORE	AUC
5-Random net	0.684 ± 0.19	0.690 ± 0.32	0.540 ± 0.470	0.658 ± 0.38	0.670 ± 0.23
gated ABMIL	0.745 ± 0.11	0.795 ± 0.20	0.673 ± 0.2	0.728 ± 0.20	0.845 ± 0.11
ABMIL	0.762 ± 0.10	0.777 ± 0.21	0.725 ± 0.21	0.75 ± 0.19	0.844 ± 0.11
Mi-NET	0.707 ± 0.64	0.707 ± 0.18	0.619 ± 0.27	0.839 ± 0.02	0.712 ± 0.003
MI-NET	0.724 ± 0.10	0.730 ± 0.10	0.763 ± 0.17	0.746 ± 0.12	0.888 ± 0.09
MI-NET with RC	0.755 ± 0.28	$0.738\pm$0.11	0.725 ± 0.21	0.731 ± 0.14	0.855 ± 0.12
MI-NET with DS	0.734 ± 0.12	0.736 ± 0.18	0.716 ± 0.18	0.728 ± 0.18	0.847 ± 0.10
Ours (euclidean)	0.890 ± 0.07	**0.943** ± **0.08**	0.821 ± 0.16	0.877 ± 0.11	0.970 ± 0.07
Ours (siamese)	**0.910** ± **0.08**	0.931 ± 0.12	**0.869** ± **0.16**	**0.898** $\pm$ **0.14**	**0.977** ± **0.13**

The experiments were run 5 times and the average (± standard error of the mean) is reported. [bold]: Highlights the best-performing results in the respective metrics

In Table 2 we report the results when we create graphs with J=5 neighbours for the UCSB dataset. In the UCSB dataset our model when compared to the other attention models improves classification accuracy by more than 10%, demonstrating the benefits of incorporating contextual information. The siamese K-MIL model outperforms its siamese counterpart, verifying the usefulness of the siamese network that manages to embed pattern similarities on top of the spatial ones.

**Fig. 5:**
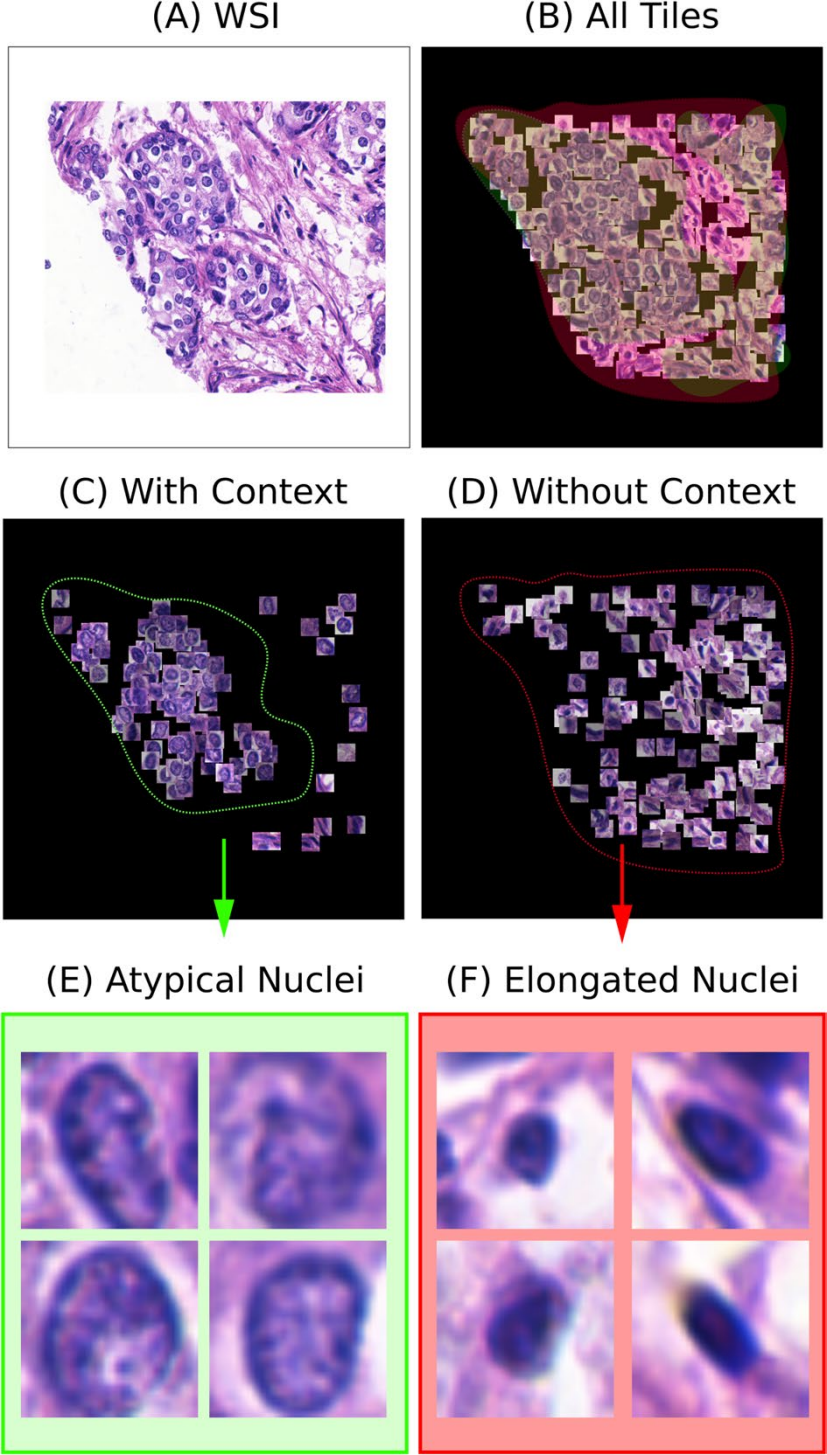
**A** WSI.** B** Cells segmented from the WSI.** C** Attention map generated by the context model.** D** Attention Map Generated by non-Context model.** E** Image highlighting atypical cells that the context model recognises as important.** F** Image of cells deemed important by the non-context model, likely reflecting normal or less relevant cell features (elongated cells)

**Fig. 6:**
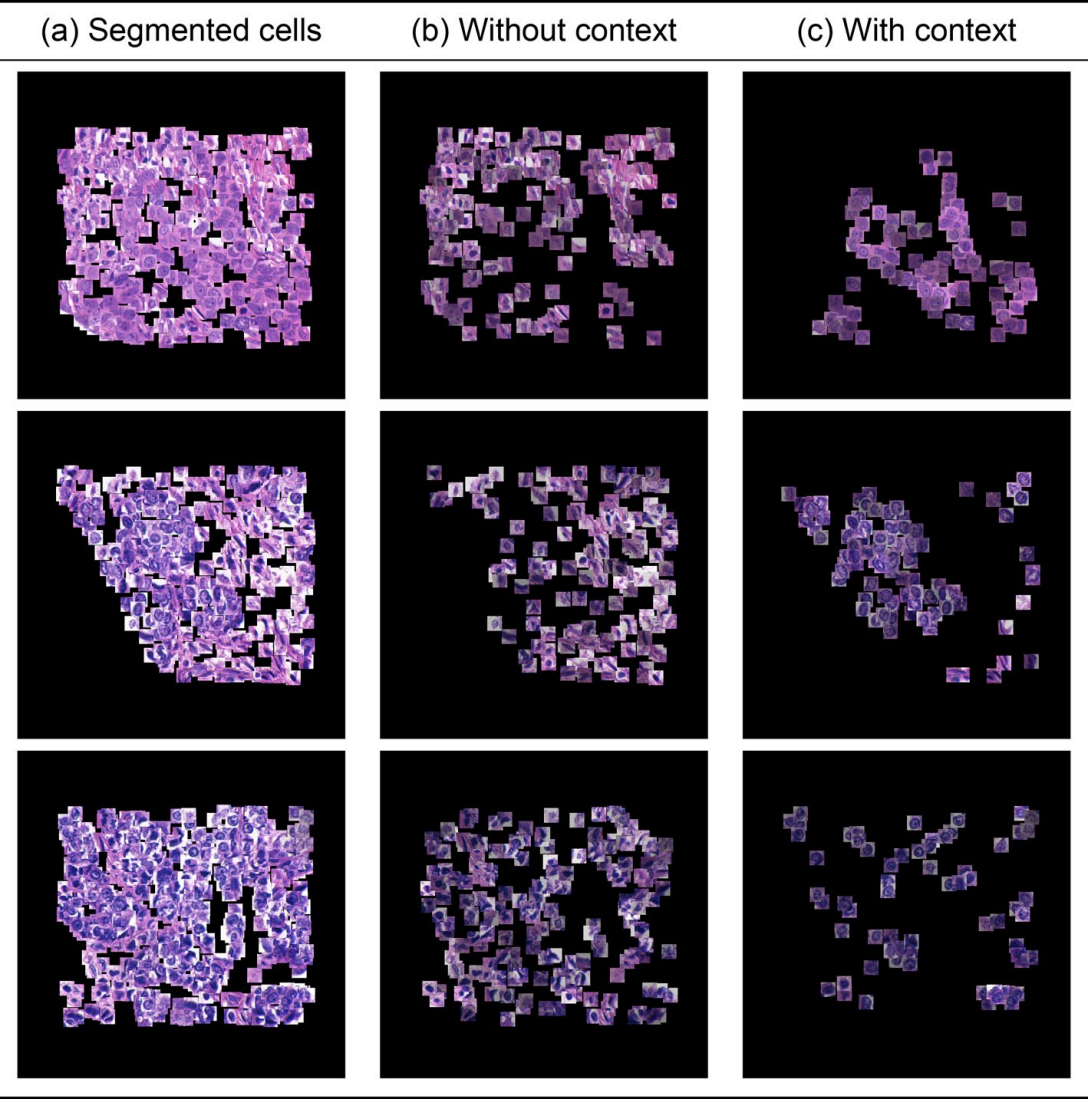
An example on the UCSB cancer dataset highlighting how our method improves instance-level classification accuracy qualitatively and quantitatively:** a** 32$\times$32 segmented cells,** b** Every patch multiplied by its corresponding attention weight using prior work [30],** c** Every patch multiplied by its corresponding attention weight using our model

In Fig. 6, we provide three representative examples of the attention maps produced by our model for the UCSB dataset. The layout of this Figure follows the same guidelines as that of Fig. 2 except it omits a column for displaying the ground truth cells, which are not available. One notable observation is that, despite the lack of ground truth labels and the predominance of cancer cells in malignant cases, ABMIL classifies all cells as important, showing no selectivity. On the contrary, the attention maps produced by K-MIL highlight cells that exhibit signs of atypia, such as irregular shapes and large, variable nuclei, which are indicative of their pathological nature as presented in Fig. 5.

**Fig. 7:**
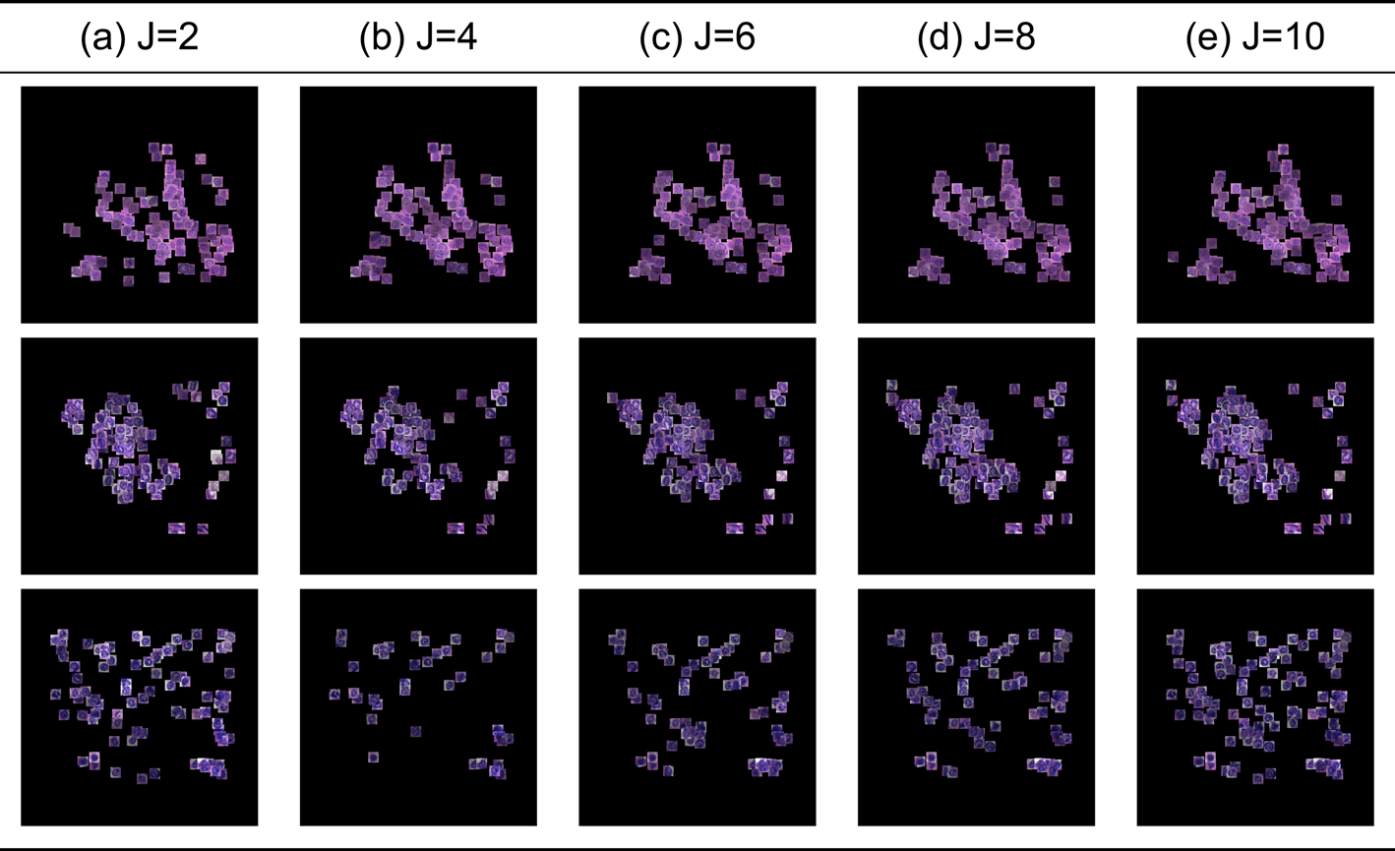
Attention map for different K on the UCSB dataset:** a** J=2,** b** J=4,** c** J=6,** d** J=8,** e** J=10. Small variations of K do not lead to drastically different results. However, as K keeps increasing, the performance drops

In Fig. 7 we showcase the attention maps produced for different variations of J for the UCSB dataset. In the case of the USCB dataset, ground truth labels are not available. A first notable observation is the increasing density of the attention maps when increasing the number of neighbours. Similarly to the colon cancer dataset, there is a risk that increasing J might lead the model to incorporate noise as a significant signal, compromising the model’s specificity.

**Fig. 8:**
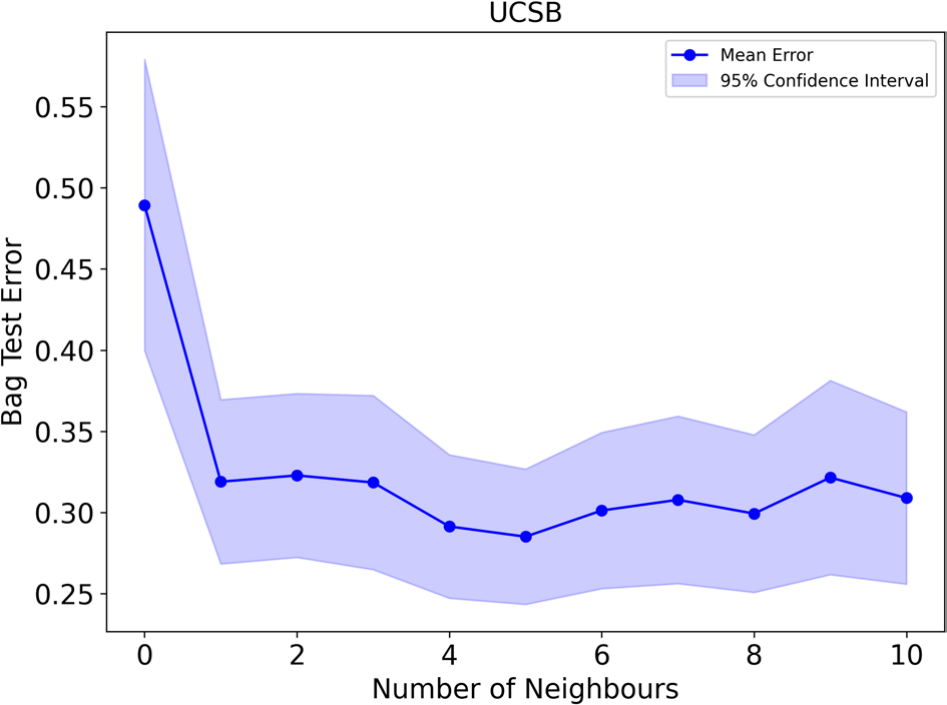
Test error with respect to the number of nodes for the UCSB cancer dataset. Each point on the plot represents the mean test error for a specific number of nodes, while the shaded regions around the points indicate the 95% confidence intervals

In Fig. 8, we present the relationship between the test error of our model and the number of neighbours considered. Again an observable trend is the reduction in bag test error as the number of nodes increases. In contrast to the COLON cancer dataset, the increase in test error as J increases is more gradual, suggesting a less pronounced impact of additional nodes on model accuracy. A small J might lead the model to make decisions based on too local a neighbourhood, possibly catching noise rather than signal. A slightly larger J, but still within a sensitive range like 2 to 5, might help the model to better generalize by considering a broader yet still relevant context of cellular features.

## Discussion

Our analysis reveals several key findings regarding the performance and adaptability of MIL models in recognising Regions of Interest (ROIs) in complex datasets. Firstly, it’s evident that MIL models lacking attention mechanisms are less flexible, hindering their ability to accurately identify ROIs. This limitation points to the critical role of attention mechanisms in enhancing model adaptability and precision. Secondly, models incorporating contextual information significantly outperform those without such integration, suggesting a topological order within the instances. This insight underscores the importance of including topological knowledge in the model, as it contributes to a more accurate representation and understanding of the data structure.

The application of the siamese network architecture demonstrates the usefulness of embedding pattern similarities alongside spatial relationships. This dual focus not only enhances the model’s performance but also its ability to discern subtle variations within the data. Furthermore, it is noteworthy that the siamese network variant of our model achieves the highest recall rates on both datasets examined. Recall is of paramount importance in the context of histopathology image analysis, where the cost of falsely classifying a positive case as negative could have serious implications for patient treatment and prognosis. This outcome highlights the siamese network’s utility in capturing critical features that contribute to more reliable and accurate classification performance.

Our neighbour analysis also reveals that there is an optimal range for the number of neighbours in graph construction that contributes to improved model performance and balances the benefits of contextual awareness against the risk of noisy inputs. Gradually increasing the number of nodes taken into account leads to enhanced model performance, as evidenced in both our quantitative and qualitative analyses. Expanding the field-of-view of each cell, which is substantiated by increasing the number of nodes used in the graph construction, provides a richer contextual understanding of the cellular environment. However, as the number of K keeps increasing (K>8), the performance of our model eventually drops. This decline suggests the introduction of noise into the model’s inputs, which likely stems from the diminished relevance of distant neighbours. As the number of nodes increases past the optimal range, the probability that these additional cells contribute meaningful contextual information decreases, and they instead begin to clutter the model’s perception with irrelevant data.

Finally regarding the computational complexity of our model, it is primarily determined by the graph attention mechanism, which involves the linear transformation of the initial feature vector of dimension $d$ into a new feature vector of dimension $d'$ for each node of the graph, resulting in $O(N \cdot d \cdot d')$ operations, where $N$ corresponds to the number of nodes in the graph.

In the Euclidean version of our model, neighbourhood connections are defined solely based on spatial proximity. After the initial linear transformation, attention scores are computed directly between spatial neighbors, without the need for additional feature-based similarity calculations. The complexity of calculating attention scores among neighbours is therefore proportional to the number of neighbours $k$ per node, leading to an overall complexity of $O(N \cdot d \cdot d') + O(N \cdot k \cdot d')$. The first term corresponds to the initial feature transformation, while the second captures the complexity of calculating the attention scores among $k$ neighbours of each node without considering feature similarity. Increasing the number of neighbours $k$ increases the number of attention scores that need to be computed, but it does not introduce additional quadratic terms. As a result, the complexity increases linearly with $k$.

In contrast, the Siamese version incorporates real-time feature similarity calculations among neighbors. After the initial linear transformation, the model computes cosine similarity scores between the feature vectors of neighboring nodes. If each node has, on average, $k$ neighbors, then for each node, the pairwise similarity among neighbors involves $k^2$ operations, resulting in a time complexity of $O(N \cdot d \cdot d') + O(N \cdot k^2 \cdot d')$. The first term corresponds to the initial feature transformation, and the second captures the complexity of calculating cosine similarity among $k$ neighbors of each node. While the linear transformation step has a complexity of $O(N \cdot d \cdot d')$, the additional term $O(N \cdot k^2 \cdot d')$ introduces a significant overhead as the number of neighbors $k$ increases. This quadratic dependency on $k$ makes it crucial to carefully choose the number of neighbors to balance between accuracy and computational efficiency. As $k$ increases, the complexity of cosine similarity computation grows quadratically, i.e., $O(k^2)$, because we need to compute similarities among all pairs of neighbors. For small values of $k$, this term $O(N \cdot k^2 \cdot d')$ may be negligible compared to $O(N \cdot d \cdot d')$. However, as $k$ becomes larger, the $O(N \cdot k^2 \cdot d')$ term can dominate, making the method computationally expensive.

## Conclusions

Our findings highlight the pivotal role of attention mechanisms, contextual integration, and optimal graph construction in improving the performance of MIL models for identifying ROIs in histopathology datasets. Models lacking attention mechanisms demonstrate limited adaptability, as they struggle to accurately identify key features. The integration of contextual information and topological order enhances representation and understanding of cell structures. Additionally, our analysis of graph construction reveals an optimal range for the number of neighbors, balancing contextual richness against the risk of noise.

## Supplementary Information

Supplementary Material 1.

## Data Availability

The COLON cancer dataset contains 100 H&E-stained histology images (bags) of colorectal adenocarcinomas. These were cropped from 10 whole-slide images of 9 patients, with a resolution of 0.55 $\upmu \hbox {m}$/pixel at 20$\times$ magnification. Each bag includes manually annotated 27 $\times$ 27 pixel nuclei classified as epithelial, inflammatory, fibroblast, or miscellaneous.The dataset used in this study is available upon request from the University of Warwick. The UCSB dataset comprises 58 H&E-stained image samples (26 malignant and 32 benign) collected from breast cancer patients. The original image dimensions are 896 $\times$ 768 pixels. This dataset is publicly available at https://bioimage.ucsb.edu/research/bio-segmentation. Project name: KMIL_BMC. Project home page: https://github.com/olgarithmics/KMIL_BMC.git. Operating system(s): Linux. Programming language: Python. Any restrictions to use by non-academics: None.

## Abbreviations

AUC: Area Under a Curve
DL: Deep Learning
DS: Deep Supervision
MIL: Multiple Instance Learning
ML: Machine Learning
MLP: Multi-Layer Perceptron
RC: Residual Connection
ROI: Region of Interest
WSI: Whole Slide Image
WSL: Weakly Supervised Learning
