# Supplementary material for: Not seeing the trees for the forest. The impact of neighbours on graph-based configurations in histopathology
Source: BMC Bioinformatics. 2025 Jan 11;26:9. doi: 10.1186/s12859-024-06007-x (PMC11724494; doi:10.1186/s12859-024-06007-x)
Supplement: Supplementary file 1 — Supplementary Material 1. [file 12859_2024_6007_MOESM1_ESM.pdf]

Supplementary Information

Experimental Settings

To enable a fair comparison between the different approaches, a common-evaluation methodology was adopted. For the COLON cancer and UCSB histopathology datasets 10-fold and 4-fold cross validation were used respectively.

Implementation Details

In our experiments the attention network as well as the Siamese network is a three-layer convolutional network that is inspired by the model introduced by (Sirinukunwattana et al. [32]) and are trained using Adam optimization algorithm (Kingma and Ba [35]). Finally, to create training pairs able to capture rich affinity relations in the case of the Siamese network we paired each instance with its two closest ones. To construct the negative pairs an equal number of non-neighbouring instances was selected.

Table 3. Training details

DATA SET	OPTIMIZER	$\beta1$, $\beta2$	LEARNING RATE	WEIGHT DECAY	STOPPING CRITERIA
COLON CANCER	ADAM	0.9,0.999	0.0001	0.0001	LOWEST VALIDATION ERROR
UCSB	ADAM	0.9,0.999	0.0001	0.0001	LOWEST VALIDATION ERROR

Table 4. Training details of the siamese network

DATA SET	OPTIMIZER	$\beta1$, $\beta2$	LEARNING RATE	WEIGHT DECAY	m_{neg}, m_{pos}	RADIUS R (PIXELS)	STOPPING CRITERIA
COLON CANCER	ADAM	0.9,0.999	0.0001	0.0001	1, 0.05	20	LOWEST VALIDATION ERR
UCSB	ADAM	0.9,0.999	0.0001	0.0001	1, 0.05	30	LOWEST VALIDATION ERR

Training Details

In every training iteration we perform data augmentation to prevent overfitting, by arbitrarily rotating patches (0°, 90°, 180°, 270°) and flipping them along vertical or horizontal axis to alleviate the rotation-variant problem of the input features. Lastly, we perform color normalization on every patch, using the the method of (Reinhard et al. [36]).
